# Feasibility of using fNIRS to explore motor-related regional haemodynamic signal changes in patients with sensorimotor impairment and healthy controls: A pilot study

**DOI:** 10.3233/RNN-221292

**Published:** 2023-12-08

**Authors:** Lina Bunketorp Käll, Malin Björnsdotter, Johanna Wangdell, Carina Reinholdt, Robert Cooper, Simon Skau

**Affiliations:** a Center for Advanced Reconstruction of Extremities (C.A.R.E.), Sahlgrenska University Hospital, Mölndal, Sweden; b Institute of Neuroscience and Physiology, Sahlgrenska Academy, University of Gothenburg, Gothenburg, Sweden; c Department of Hand Surgery, Institute of Clinical Sciences, Sahlgrenska Academy, University of Gothenburg, Gothenburg, Sweden; d Department of Medical Physics and Biomedical Engineering, Biomedical Optics Research Laboratory, University College London, UK

**Keywords:** Spinal cord injury, tendon transfer, plasticity, motor cortex, functional near-infrared spectroscopy

## Abstract

**Background:**

While functional near-infrared spectroscopy (fNIRS) can provide insight into cortical brain activity during motor tasks in healthy and diseased populations, the feasibility of using fNIRS to assess haemoglobin-evoked responses to reanimated upper limb motor function in patients with tetraplegia remains unknown.

**Objective:**

The primary objective of this pilot study is to determine the feasibility of using fNIRS to assess cortical signal intensity changes during upper limb motor tasks in individuals with surgically restored grip functions. The secondary objectives are: 1) to collect pilot data on individuals with tetraplegia to determine any trends in the cortical signal intensity changes as measured by fNIRS and 2) to compare cortical signal intensity changes in affected individuals versus age-appropriate healthy volunteers. Specifically, patients presented with tetraplegia, a type of paralysis resulting from a cervical spinal cord injury causing loss of movement and sensation in both lower and upper limbs. All patients have their grip functions restored by surgical tendon transfer, a procedure which constitutes a unique, focused stimulus for brain plasticity.

**Method:**

fNIRS is used to assess changes in cortical signal intensity during the performance of two motor tasks (isometric elbow and thumb flexion). Six individuals with tetraplegia and six healthy controls participate in the study. A block paradigm is utilized to assess contralateral and ipsilateral haemodynamic responses in the premotor cortex (PMC) and primary motor cortex (M1). We assess the amplitude of the optical signal and spatial features during the paradigms. The accuracy of channel locations is maximized through 3D digitizations of channel locations and co-registering these locations to template atlas brains. A general linear model approach, with short-separation regression, is used to extract haemodynamic response functions at the individual and group levels.

**Results:**

Peak oxyhaemoglobin (oxy-Hb) changes in PMC appear to be particularly bilateral in nature in the tetraplegia group during both pinch and elbow trials whereas for controls, a bilateral PMC response is not especially evident. In M1 / primary sensory cortex (S1), the oxy-Hb responses to the pinch task are mainly contralateral in both groups, while for the elbow flexion task, lateralization is not particularly clear.

**Conclusions:**

This pilot study shows that the experimental setup is feasible for assessing brain activation using fNIRS during volitional upper limb motor tasks in individuals with surgically restored grip functions. Cortical signal changes in brain regions associated with upper extremity sensorimotor processing appear to be larger and more bilateral in nature in the tetraplegia group than in the control group. The bilateral hemispheric response in the tetraplegia group may reflect a signature of adaptive brain plasticity mechanisms. Larger studies than this one are needed to confirm these findings and draw reliable conclusions.

## 1 Background

The human brain is controlled through large-scale neural networks between cortical regions of which the primary motor cortex (M1) is the principal area involved in controlling volitional movements (Chiou et al., 2013; Heming et al., 2019). Unilateral body movements are mainly controlled by contralateral cortical regions (Lee et al., 2019; Nardone et al., 2013; Yang et al., 2020; Yokoyama et al., 2019). However, the presence of ipsilateral cortical involvement in unimanual tasks has been repeatedly demonstrated in previous studies (Bruurmijn et al., 2021; Buetefisch et al., 2014; Bundy and Leuthardt, 2019; Chen et al., 1997; Chiou et al., 2013; Heming et al., 2019; Matsumoto et al., 2021; Schwartz, 2016; Wisneski et al., 2008). Nevertheless, the extent or magnitude of this relationship has not been definitively established (Derosiere et al., 2014), due in part to discrepancies among studies in the time course and localization of activity (Huang et al., 2004). As an example, it has been demonstrated that unilateral finger movement produces ipsilateral activation in the premotor area rather than in M1 (Cramer et al., 1999; Huang et al., 2004), which is consistent with the findings of another study (Dassonville et al., 1998).

Previous findings suggest that the ipsilateral motor cortex activation in unilateral motor tasks may reflect part of a bilateral network involved in the planning and/or execution of volitional motor tasks in the ipsilateral hand (Bundy and Leuthardt, 2019; Derosiere et al., 2014; Horenstein et al., 2009). Previous neuroimaging studies point to a role of the ipsilateral motor and premotor cortex in complex and highly precise tasks as well as in movements that require multiple joint coordination (Barany et al., 2020; Verstynen et al., 2005; Wilkins and Yao, 2020). A growing volume of research suggests that the compensatory cerebral mechanisms of volitional motor control following a stroke involve increased activation of ipsilateral cortical regions (Askim et al., 2009; Favre et al., 2014; Johansen-Berg et al., 2002; Van Dokkum et al., 2018; Werhahn et al., 2003). Similarly, it has been proposed that the ipsilateral M1 plays a significant role in compensating for functional deficits and mediating functional motor recovery in paretic hands after cervical spinal cord injury (SCI) (Lundell et al., 2011; Nardone et al., 2013).

A large number of neuroimaging studies have been conducted to explore the neural basis of motor performance by means of a variety of techniques, such as functional magnetic resonance imaging (fMRI), electroencephalography (EEG) and transcranial magnetic stimulation (TMS). Functional near-infrared spectroscopy (fNIRS) has emerged as a valid and particularly accessible neuroimaging modality that enables researchers to investigate cerebral oxygenation in response to various stimuli such as motor execution (Derosiere et al., 2014; Herold et al., 2017; Shibuya and Kuboyama, 2007; Shibuya et al., 2008). As with fMRI, fNIRS measures the relative concentration of oxygenated and deoxygenated haemoglobin (oxy-Hb/deoxy-Hb) and thus has the potential to elucidate some key issues concerning the basis of vascular response (Steinbrink et al., 2006). The emergence of fNIRS has increased the flexibility of measurements with respect to the populations and the specific behaviours that can be investigated (Leff et al., 2011).

We conducted a pilot study to determine the feasibility of using fNIRS to assess cortical signal intensity changes during upper limb motor tasks in individuals with surgically restored grip functions. The secondary objectives were 1) to collect pilot data on individuals with tetraplegia to determine any trends in cortical signal intensity changes as measured by fNIRS and 2) to compare cortical signal intensity changes in affected individuals versus age-appropriate healthy volunteers. Specifically, the patients in our study presented with tetraplegia, a type of paralysis resulting from a cervical SCI causing loss of movement and sensation in both lower and upper limbs. All patients had their grip functions restored by surgical tendon transfer, a procedure which constitutes a unique, focused stimulus for brain plasticity (Bunketorp Käll et al., 2018).

## 2 Methods

### 2.1 Study design and participants

fNIRS was used to assess oxy-Hb signal changes of cortical activation during the performance of two right-sided motor tasks in patients with sensorimotor impairment and in healthy controls. The study included six right-handed males with tetraplegia and a mean age of 40 (range: 31–48) who underwent grip reconstruction surgery on their dominant right hand between 2005 and 2014 at Sahlgrenska University Hospital in Gothenburg, Sweden. By transferring the brachioradialis (BR) tendon to the paralyzed right thumb flexor, the flexor pollicis longus (FPL) and original function (thumb flexion) was restored. A gender- and age-matched control group, all right-handed, was recruited. The control group had a mean age of 39 (range: 29–46). Eligibility criteria were: 1) the surgery must have been performed at least one year prior to inclusion and must have included restoration of thumb flexion with the goal of reconstructing an active key pinch by a BR-to-FPL tendon transfer. More than one tendon transfer performed to restore key pinch caused exclusion. Other tendon transfer procedures on the same upper limb were allowed; however, 2) no motor function below the wrist, such as on the finger and/or thumb extensors prior to surgery, 3) complete or incomplete SCI with an injury level C4–C7 with intact BR control prior to surgery, 4) no history of medical or other neurological diseases that might affect the investigated parameters, 5) no defective vision that requires the use of glasses in the fMRI assessment, 6) individual factors that preclude entering the fMRI environment (e.g. metal implants which are not compatible with the MRI environment, pacemakers and claustrophobia), 7) ability to speak and understand Swedish, and 8) ability to travel to Gothenburg. Prior to inclusion, all participants received oral and written information about the study procedures. Informed consent from all study participants was obtained. Moreover, the study was approved by the Regional Ethical Review Board, Gothenburg, Sweden (Dnr: 679-15).

### 2.2 Motor paradigm

**Fig. 1:**
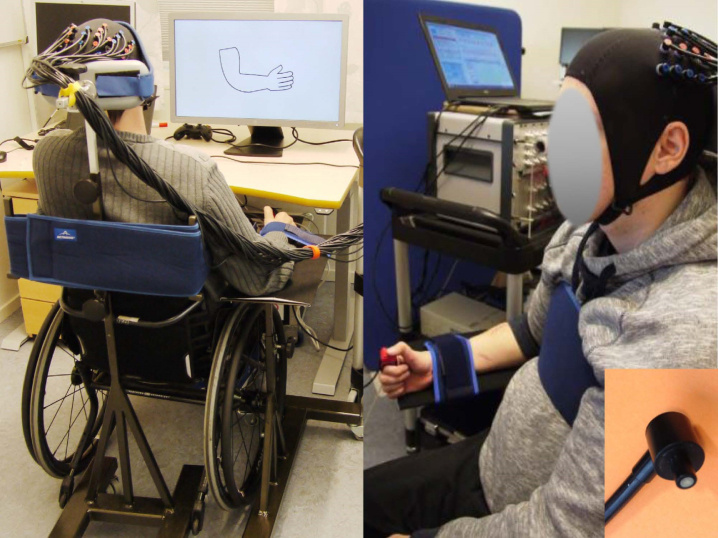
fNIRS experimental set-up. Foam-covered pads were used to apply the specially designed adult optical fibers firmly but comfortably to the subjects’ heads. Subjects were seated with the forearm supported on an adjustable table, and the wrist strapped. Due to very limited or complete loss of trunk stability in patients, a specially designed and portable neck rest was constructed that the subjects were strapped towards, with the aim of minimizing movement of the torso. A small cube was attached to the index finger to keep the thumb stabilized and somewhat elevated in relation to the index finger. Permission was granted by the patient.

For each subject, 6 blocks consisting of a series of 10 movement trials were performed. The inter-stimulus interval (ISI) was 12–15 s long and was pseudo-randomized as was the order of conditions, allowing for reduced anticipation effects and minimized synchronization between motor execution and Mayer waves. Each trial was 10 s, and the duration of each block thus lasted approximately 250 s. Within a block, two motor tasks, *key pinch and elbow flexion*, were presented on a screen, and both tasks were repeated five times in every block in pseudorandomized order. Isometric activation of the motor tasks was chosen to make the motor execution as consistent and well-defined as possible as well as to minimize movement artifacts. Across all blocks, each movement type was repeated 300 times. Participants were given visual cues on the screen: blinking graphic illustrations depicting either a key pinch or an elbow (Fig. 1) paced by digitized sound effects. Each of the 2 motor tasks lasted 1 s, a speed that was found to be easy and comfortable to perform in pilot trials. Participants executed the tasks once every second during each trial, after which the illustration was replaced by a central black circle (of one size). The circle blinked once a second for 10–15 s, and subjects were instructed to visualize the fixation circle and remain motionless. One of two different digitized sounds signifying each of the two conditions was played in synchrony with the last circle flash, indicating the movement to be executed in the next trial. During the ISIs, subjects were instructed to visualize a fixation ring and remain motionless. Between blocks, subjects were reminded to avoid any movements not associated with the required tasks.

### 2.3 fNIRS experimental setup

The fNIRS assessment was conducted in a quiet and darkened room at MedTech West of Sahlgrenska University Hospital, Gothenburg, Sweden. Data was collected using an NTS Optical Imaging System (University College London). During the fNIRS measurement, all subjects were seated with their right shoulders in a neutral position, the elbow in 60° of flexion (Boland et al., 2008), the forearm supported on an adjustable table and the wrists strapped to minimize motion artifacts (Fig. 1). A small plastic cube was attached to the index finger using a small strip of Velcro tape to keep the thumb stabilized and somewhat elevated in relation to the index finger (Fig. 1). Due to the greatly limited or complete loss of trunk stability after cervical SCIs, a specially designed neck rest was constructed to enable strapping the participant’s head and torso, with the aim of minimizing movement when executing motor tasks (Fig. 1). Individuals with tetraplegia remained seated in their wheelchairs. The participants were instructed to avoid any movements other than those needed for the two motor tasks. The participants were given the opportunity to briefly rehearse the tasks before starting the assessment to ensure that they comprehended the task instructions displayed on the computer screen. Prior to the experiment, the maximal voluntary contraction (MVC) during isometric activation of the key pinch was measured for each participant. All subjects were then trained to exert target forces corresponding to approximately 20% of the MVC, which was the target force in the fNIRS assessment.

### 2.4 fNIRS data acquisition

**Fig. 2:**
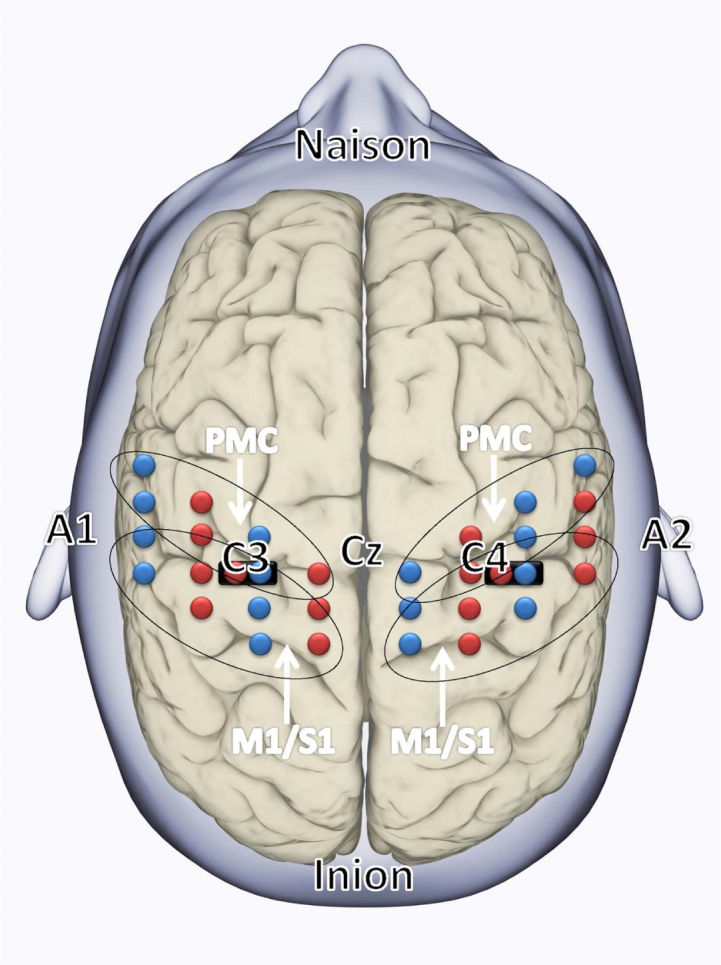
The fNIRS probe set was positioned over the motor areas. Black ovals indicate the covered areas of the sensory-motor regions of the left and right hemispheres: M1/S1 (primary motor/sensory cortex) and premotor cortex (PMC). The fNIRS array consisted of 16 sources (red) and 32 detectors (blue) with the short distance source-detector pairs depicted with black markings. The EEG International 10/20 system, including Cz, naison, inion, A1, A2, C3, and C4 locations are indicated.

**Fig. 3:**
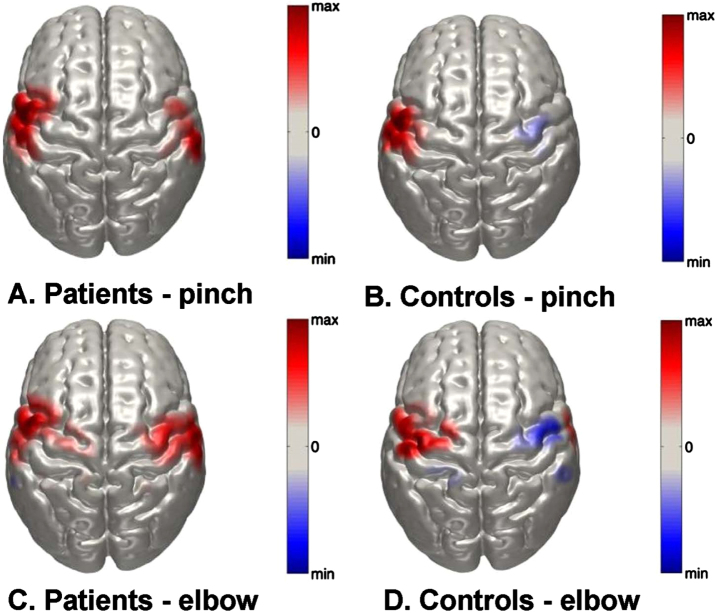
Normalized group-level DOT images of oxy-Hb responses in the right and left hemispheres in response to right sided A) key pinch task in patients; B) elbow-flexion task in patients; C) key pinch task in control subjects and D) elbow-flexion task in control subjects. Scale is arbitrary.

The fNIRS system consists of two types of optical probes (or optodes): the light emitting sources that send infrared light that penetrates the skull and cerebral cortex, and the light detectors that collect a proportion of that light which is reflected to the scalp. The probes were fixed to the subjects’ heads using specially designed, foam-covered optode holders attached to an Easycap. The adult probes were designed in such a way that they had optimal contact with the scalp (Fig. 1). To ensure optimal signal quality, a hairpin was used to displace hair from the channel centre. A multiplexing scheme was used. In total, there were 16 sources and 16 detector fibre bundles covering the sensorimotor cortical regions. We defined a region of interest (ROI) including the primary motor/sensory cortex (M1/S1) and premotor cortex (PMC) as indicated in Fig. 2. Source–detector pairs were positioned over the sensory–motor regions of the left and right hemispheres, with the covered area corresponding to the C3 and C4 positions in the EEG International 10/20 system. To ensure the fNIRS probes were optimally positioned over M1, four anatomic landmarks in the EEG International 10/20 system were used to position the Easycap: the nasion, inion, A1 and A2 (Fig. 3). The specific Brodmann areas representing each cortical region of interest are M1/S1: primary sensorimotor cortex (S1: Brodmann Area 1, 2 and 3; M1: Brodmann Area 4) and PMC: premotor cortex (Brodmann Area 6).

Near-infrared light with wavelengths of 780 and 850 nm was delivered from the fNIRS system to measure the concentration changes of oxygenated haemoglobin (oxy-Hb) and deoxygenated haemoglobin (deoxy-Hb) in the cerebral cortex. The reflected light was sampled at a frequency of 10 Hz using a multiplexing approach, where each source was coupled with up to 6 detectors with separations ranging from 1 to 5 cm, resulting in a total of 83 source–detector pairs (called channels) for each wavelength. In order to filter out superficial background haemodynamics unrelated to voluntary motor activation, 2 short (1.5 cm) separation channels were used, as depicted in Fig. 2. This array enabled recordings of cortical activation at a relatively high spatial resolution. A Polhemus Patriot system was employed to digitize the optode locations and cranial landmarks for each subject. To visualize the group-level functional responses of our cohorts, we registered the digitized optode positions to a finite element mesh based on the MNI152 atlas (Dempsey et al., 2015). Diffuse optical tomography (DOT) images of the oxy-Hb functional responses were then reconstructed. Then we built a subject-specific forward model using the TOAST++light transport modelling package (Schweiger and Arridge, 2014) and constructed images in the full 3D volume of the head mesh. Individual-level images were projected to the cortical surface mesh of the same head model to allow for averaging and visualization.

### 2.5 fNIRS data analysis

The fNIRS data were preprocessed using MATLAB 2018b (MATLAB. (2018). version 9.5.944444 (R2018b). Natick, Massachusetts: The MathWorks Inc) and the MATLAB-based fNIRS-processing package HomER3 (Huppert TJ, 2009). The processing pipeline started with pruning of raw data. Channels were rejected if their mean intensity was below 1e-3 arbitrary units. The raw data were then converted to optical density (OD). A low band-pass 0.5 filter was used to remove pulse and respiration. The HomER3 functions hmrMotionArtifact and hmrMotionCorrectSpline were used to correct motion artifacts. OD was converted to haemoglobin concentration by the function hmrR_OD2Conc, where the differential path length was chosen based on age (Scholkmann and Wolf, 2013).

To calculate the haemodynamic response function (HRF), the hmrR_GLM function in HomER3, which estimates the HRF by applying a general linear model (GLM), was used. To solve the GLM, a least-square fit of a convolution model (Ye et al., 2009) was utilized. In the model, the HRF at each channel and chromophore was modelled through a modified gamma function using recommended values for tau and sigma (0.1 and 3.0 for oxy-Hb and 1.8 and 3.0 for deoxy-Hb). The GLM also included polynomial drift regressors up to the third order. The regression time length was -5 to 20 s. The short separation channel selection for regression of each long channel was chosen based on the highest correlation to the long channel. To visualize the spatial distribution of the group-level responses, we calculated the average of cortical reconstructions for all subjects over a 5 s temporal window centred at the 10 s post-trial onset. This average cortical oxy-Hb image was normalized for each subject (such that every value in the image was divided by the maximum absolute value of that image) to remove the individual effects of the response scale prior to group averaging.

### 2.6 Statistics

We used the open-source program JASP version 0.16.4 (Marsman and Wagenmakers, 2017) for data analyses. The statistic used for the fNIRS data was the mean beta for oxy-Hb from several channels pooled together into the predefined regions of interest (ROIs) PMC and M1/S1, as shown in Fig. 2. We did two repeated measures ANOVAs, one for each ROI, with the between-subject factor *Group* (controls and patients) and within-subject factor *Task* (pinch and elbow flexion) and *Hemisphere* (contralateral and ipsilateral).

Since this was a pilot study that involved a few participants and was aimed at assessing feasibility, an exploratory method of analysis was used. Therefore, no conventional null hypothesis significance testing was done, where an alpha value was set to control the error rate. Instead, we used a Fisherian interpretation of p-values (Perezgonzalez, 2015), where a low p-value was interpreted as indirect inductive evidence against the null hypothesis (here defined as no difference). In contrast to the confirmatory analysis, no claims regarding type 1 error rate are made here and no correction for multiple comparisons is performed. Under a Fisherian interpretation, it is possible to use a threshold for ‘significance’. However, since this terminology is intimately connected to type 1 error control under the null hypothesis significance testing framework and might lead to confusion, we did not apply the term ‘significance’ to our exploratory analysis.

## 3 Results

### 3.1 Participant demographics

The average number of years that elapsed since the patients underwent surgery was 7 (range: 2–10). The mean age at the time of surgery was 34 years (range: 29–43). The neurological level of SCI was graded as C6 in 2 participants and C7 in 3 participants. The description of motor groups according to the International Classification for Surgery of the Hand in Tetraplegia (McDowell et al., 1979) was graded 4 in 3 participants and 3 and 2 in the remaining 2. The causes of cervical SCI among participants were a diving accident (*N* = 2), motocross (*N* = 1), occupational injury (*N* = 1), and a falling accident (*N* = 1). A summary of all surgical procedures is presented in Table 1. A gender and age-matched control group was recruited. All the members of the control group were right-handed and had a mean age of 39 (range: 29–46).

**Table 1:** Demographics, clinical characteristics, and surgical procedures included among the individuals with tetraplegia

Patient	Age	Time since surgery	Cause of injury	BR function (0 - 5) ^1^	International Classification^2^	Level of injury	Surgical procedures
1	31	1	Diving	5	4	C7	tf, ff, ir, fpl-epl, elk, ecu, cmcI
2	41	10	Fall	5	3/4	C6	tf, ff, ir, fpl-epl, cmcI
3	48	5	Work-related	5	4	C7	tf, ff, ir, fpl-epl, ecu, cmcI
4	39	7	Sport	5	2	C6	tf, fpl-epl, elk, cmcI
5	41	10	Traffic	5	2	C6	tf, ff, fpl-epl, ir
6	43	7	Diving	5	4	C7	tf, ff, ir, fpl-epl, cmcI, ecu

1) Classified according to the Medical Research Council (MRC) system; 2) Description of motor groups according to the International Classification for Surgery (ICSHT) of the Hand in Tetraplegia; BR = brachioradialis; tf = thumb flexion reconstruction; ff = finger flexion reconstruction; ir = intrinsic reconstruction; elk = Extensor pollicis longus-loop-knot; fpl-epl=Split FPL - EPL tenodesis; ecu = Extensor Carpi Ulnaris tenodesis; cmcI = arthrodesis of carpometacarpal (CMC) joint I.

### 3.2 fNIRS result

Figure 3 represents DOT images of peak oxy-Hb changes in the right and left hemispheres (both ROIs combined) in response to the two motor tasks. Figure 3A and C depict the peak oxy-Hb response to the key pinch and elbow flexion tasks in the tetraplegia group. The peak oxy-Hb changes during both tasks appear to be highly bilateral in nature. In contrast, Fig. 3B and D show equivalent maps in the control group that have a distinct lateralization pattern. Furthermore, the elbow task shows a peak response that is seemingly superior to that of the key pinch task.

**Fig. 4:**
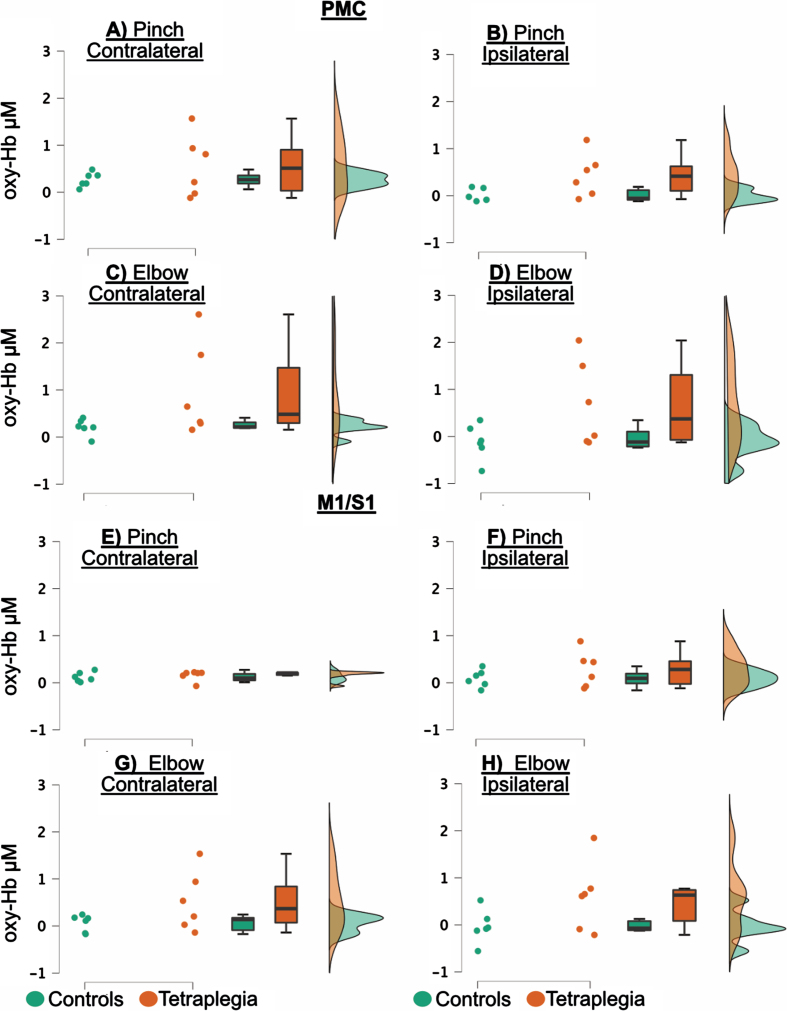
Raincloud plots of oxy-Hb, visualizing each data point (dots), boxplots and data distributions. Controls are in green and patients in red. Figure 4A–D are premotor cortex (PMC) and figures E–H are primary motor/sensory cortex (M1/S1). The Y-axis variable is micro molar (μM).

In Fig. 4, raincloud plots of oxy-Hb are presented to visualize each data point (dots), boxplots and distribution of data. For the tetraplegia group, the functional activity was highly variable for both tasks apart from the hemispheric response in the contralateral hemisphere during the pinch task which was homogeneous. Overall, the raincloud plots demonstrate seemingly higher oxy-Hb responses in the tetraplegia group than in the controls. Further, Fig. 4 shows that activation in the ipsilateral hemisphere was primarily present for the tetraplegia group (Fig. 4B, D, F and H), in contrast to the controls (figures A, C, E and G).

The repeated measures ANOVA showed a *Group* difference in which patients had an increased functional activity compared to control subjects. The *Group* difference was significant in the PMC region, with F(1,10)=5.134, *p* = 0.047 and η^2^_p_ =0.339 (Fig. 4A–D). In the M1/S1 region, F(1,10)=3.708, with *p* = 0.083 and η^2^_p_ = 0.149 (Fig. 4E–H), respectively. There was a *Hemisphere* difference in PMC, with F(1,10)=6.335, *p* = 0.031 and η^2^_p_ =0.388, where there was overall high activity in the contralateral cortex (Fig. 4A–D), but not in M1/S1, with F(1,10)=6,335, *p* = 0.031 and η^2^_p_ =0.388 (Fig. 4E–H). No other difference was detected either for the *Task* or for any interactions (results are summarized in Table 2 and Fig. 4).

**Table 2:** Test statistics from the repeated ANOVA

	Premotor cortex			Primary sensorimotor		
	(PMC)			cortex (M1/S1)		
	F	p	η^2^_p_	F	p	η^2^_p_
Group	5.134	0.047	0.339	3.708	0.083	0.149
Task	0.341	0.572	0.033	0.898	0.366	0.021
Hemisphere	6.335	0.031	0.388	0.069	0.799	0.0007
Task vs. Group	1.100	0.319	0.099	2.726	0.130	0.064
Hemisphere vs. Group	0.215	0.653	0.021	0.893	0.367	0.010
Task vs. Hemisphere	2.170	0.171	0.178	0.829	0.384	0.001

## 4 Discussion

To the best of the authors’ knowledge, this is *the* first *study* of *its* kind to assess the feasibility of utilizing fNIRS to assess motor-related neural activity during volitional upper limb motor tasks in patients with tetraplegia who had their grip functions restored by surgical tendon transfers. The two motor tasks elicited clear activations in contralateral motor cortices of both patients and controls, similar to previous findings (Nardone et al., 2013; Yang et al., 2020), especially during tasks performed using the dominant hand (Lee et al., 2019; Yokoyama et al., 2019). The bilateral activation demonstrated for some of the patients in the present study is in accordance with the findings of previous studies (Verstynen et al., 2005; Wilkins and Yao, 2020), pointing to the role of the ipsilateral motor and premotor motor cortex in complex tasks as well as in movements that require multiple joint coordination. For the patients in the present study, the pinch task represented a re-established motor function, namely, they all had their pinch grip surgically restored by means of transferring an elbow flexor to a paralyzed thumb flexor. This transfer entailed adapting the elbow flexor to also control the thumb (Bunketorp Kall et al., 2018), a task which could initially be considered complex because it requires significant mental focus to learn new motor patterns (Wangdell et al., 2016). In addition, a well-controlled key pinch involves coordinating multiple joints given that synergistic co-contraction of triceps is necessary to avoid elbow flexion (Bunketorp Kall et al., 2018).

Given that several years had passed for most patients since they underwent surgery, their motor patterns could be considered well-established at the time of this study, enabling task execution without heightened attention. However, we cannot rule out that the bilateral hemispheric response demonstrated in the tetraplegia group may reflect a signature of adaptive brain plasticity mechanisms (Merzenich et al., 2014). The muscles acting as elbow flexors were intact in the tetraplegia group, which might explain why the group’s cortical responses during elbow flexion were similar to those of the control subjects.

The individuals with tetraplegia had a highly variable degree of oxy-Hb activation during the motor tasks. Activation in the ipsilateral hemisphere was primarily present for the tetraplegia group although it was highly variable for both tasks. The oxy-Hb analyses revealed that both the PMC and M1/S1 responses were highly variable from subject to subject. While the overall responses in M1/S1 were relatively bilateral across all individuals with tetraplegia for both tasks, some subjects demonstrated more pronounced bilateral M1/S1 activation during one unilateral task (pinch or elbow flexion) compared with the other. This variability in activation may be related to differences in noise characteristics across fNIRS sessions that affect the global level of fNIRS brain activation.

The findings of the present study suggest that increased oxy-Hb in brain regions associated with upper extremity sensorimotor processing were more bilateral in nature in the tetraplegia group than in the controls. Moreover, the individuals with tetraplegia had a seemingly high oxy-Hb response during both pinch and elbow trials, whereas controls had overall low activity.

Our findings are in accordance with those of a previous fMRI/TMS study (Chiou et al., 2013), which demonstrated that co-activation of the ipsilateral M1 was closely related to the motor executions of the contralateral M1. The ipsilateral co-activations of M1 were interpreted as intra- and interhemispheric interactions thought to be critical for unilateral motor tasks. In a review (Chettouf et al., 2020), the bilateral neural activation typically observed for bimanual movements was said to resemble the bi-hemispheric activation patterns during unimanual movements. The authors argued that the ipsilateral activation patterns during unimanual movements serve to suppress interhemispheric crosstalk through transcallosal tracts (Chettouf et al., 2020). The unique representation of ipsilateral hand movements in the human sensorimotor cortex demonstrated by means of fMRI in a previous study (Bruurmijn et al., 2021) further supports the notion of transcallosal integrative processes that support optimal coordination of hand movements.

It is unclear whether the large ipsilateral oxy-Hb response in the tetraplegia group is the result of an adaptive mechanism, meaning a large bilateral network is involved in task execution, or whether it represents transcallosal inhibitory signals aimed at suppressing unwanted movements in the ipsilateral upper limb. It is highly likely that the observed ipsilateral activity represents both inhibitory and excitatory neuronal activity including both callosally mediated interaction with the contralateral hemisphere, as previously suggested (Bruurmijn et al., 2021; Bundy and Leuthardt, 2019) as well as neural processes directly related to task execution. The authors of a previous fMRI study (Berlot et al., 2019), argued that premotor cortical activity during finger presses may reflect attentional signals. Even though restored functions were considered to be well-established for individuals with tetraplegia, motor execution may still be associated with attentional demand, resulting in preparatory signals.

There are several limitations to this study. First, the small number of participants limits the generalizability of the findings. Given interindividual variability, especially in the SCI group, large populations are needed to investigate task-related cortical activation. Second, since the study focused on the dominant side of right-handed participants, the findings could not be generalized to motor tasks performed by the non-dominant side. Neither could the findings be generalized to motor tasks performed by the left upper limb. Future studies should include left-handed participants and motor tasks performed by the non-dominant side. It is not possible to distinguish between primary motor and sensory motor cortical activity in the present study since both areas were included in one single ROI (M1/S1). However, since the importance of sensory information in feedback control has been demonstrated (Berlot et al., 2019), we chose to combine both motor and somatosensory cortical areas in the fNIRS analyses. Moreover, since fNIRS has limited spatial resolution as compared to fMRI, there is a technical difficulty in utilizing fNIRS to assess oxy-Hb changes in two closely located cortical regions such as the primary motor and sensory cortical areas.

Despite its weaknesses, this study has strengths. First is the inclusion of individual MRI data to confirm the spatial registration of each channel on the brain. Another strength is that we aimed at maximizing the accuracy of channel locations using 3D digitizations of channel locations and co-registering these locations to template atlas brains. Further, short-separation channels were used to filter out superficial background haemodynamics unrelated to voluntary motor activation. To minimize motion artifacts, we used a specially designed neck rest to which the subjects’ heads were strapped during the execution of motor tasks. Lastly, our research measured sensorimotor cortices in both hemispheres, which enabled us to examine lateralization.

The present findings showing that the ipsilateral hemisphere seems to be involved in the execution of unilateral movements have important implications for understanding motor control in unimpaired humans as well as motor-impaired populations. This study supports the feasibility of using fNIRS in researching motor-related cortical areas, enabling future researchers to investigate the neural correlates for motor control. The advantageous features of the study allow highly ecologically valid investigations that can translate laboratory work into clinical environments in motor-impaired patient populations (Irani et al., 2007).

In summary, we have demonstrated for the first time that it is feasible to use fNIRS to study haemoglobin-evoked responses during volitional upper limb motor tasks in individuals with tetraplegia who had their grip functions restored by surgical tendon transfers. Although the sample size is judged to be sufficient to determine the feasibility of using fNIRS to measure haemoglobin-evoked responses in this context, it is too small to draw any definitive conclusions. Nevertheless, the experimental setup was deemed suitable. Signal quality was satisfactory, and the findings suggest that different motor tasks elicit differential brain activity, supporting the set-up’s scientific feasibility. Besides, the results of the between-group comparisons are suggestive that cortical signal changes in brain regions associated with upper extremity sensorimotor processing are more bilateral in nature in the tetraplegia group than in the control group. This between-group finding, therefore, supports the scientific feasibility of our setup and the usability of fNIRS to investigate the neural basis of restored volitional upper limb motor control in patients with tetraplegia.

In conclusion, this pilot study showed that the experimental setup was feasible for assessing cortical haemodynamics related to voluntary upper limb motor activation using fNIRS in individuals with surgically restored grip functions. Cortical signal changes in brain regions associated with upper extremity sensorimotor processing appeared larger and more bilateral in nature in the tetraplegia group than in controls. The bilateral hemispheric response in the tetraplegia group may reflect a signature of adaptive brain plasticity mechanisms. Larger studies than the present one are needed to confirm these findings and draw reliable conclusions as well as to clarify the meanings of the oxy-Hb changes in cortical areas during motor tasks.

## References

[ref001] Askim, T. , Indredavik, B. , Vangberg, T. , Haberg, A. (2009) Motor network changes associated with successful motor skill relearning after acute ischemic stroke: a longitudinal functional magnetic resonance imaging study. Neurorehabilitation and Neural Repair, 23(3), 295–304. doi: 10.1177/1545968308322840. 18984831

[ref002] Barany, D. A. , Revill, K. P. , Caliban, A. , Vernon, I. , Shukla, A. , Sathian, K. , Buetefisch, C. M. (2020) Primary motor cortical activity during unimanual movements with increasing demand on precision. Journal of Neurophysiology, 124(3), 728–739. 32727264 10.1152/jn.00546.2019 PMC7509291

[ref003] Berlot, E. , Prichard, G. , O’Reilly, J. , Ejaz, N. , Diedrichsen, J. (2019) Ipsilateral finger representations in the sensorimotor cortex are driven by active movement processes, not passive sensory input. Journal of Neurophysiology, 121(2), 418–426. 30517048 10.1152/jn.00439.2018 PMC6397402

[ref004] Boland, M. R. , Spigelman, T. , Uhl, T. L. (2008) The function of brachioradialis. Journal of Hand Surgery, 33(10), 1853–1859. doi: 10.1016/j.jhsa.2008.07.019. 19084189

[ref005] Bruurmijn, M. L. , Raemaekers, M. , Branco, M. P. , Ramsey, N. F. , Vansteensel, M. J. (2021) Distinct representation of ipsilateral hand movements in sensorimotor areas. European Journal of Neuroscience, 54(10), 7599–7608. 34666418 10.1111/ejn.15501 PMC9297959

[ref006] Buetefisch, C. M. , Revill, K. P. , Shuster, L. , Hines, B. , Parsons, M. (2014) Motor demand dependent activation of ipsilateral motor cortex. American Journal of Physiology-Heart and Circulatory Physiology, 112(4), 999–1009. 10.1152/jn.00110.2014 PMC4122744 24848477

[ref007] Bundy, D. T. , Leuthardt, E. C. (2019) The cortical physiology of ipsilateral limb movements. Trends in Neurosciences, 42(11), 825–839. 31514976 10.1016/j.tins.2019.08.008 PMC6825896

[ref008] Bunketorp Kall, L. , Cooper, R. J. , Wangdell, J. , Friden, J. , Bjornsdotter, M. (2018) Adaptive motor cortex plasticity following grip reconstruction in individuals with tetraplegia. Restorative Neurology and Neuroscience, 36(1), 73–82. doi: 10.3233/RNN-170775. 29439365 PMC5817907

[ref009] Bunketorp Käll, L. , Cooper, R. J. , Wangdell, J. , Fridén, J. , Björnsdotter, M. (2018) Adaptive motor cortex plasticity following grip reconstruction in individuals with tetraplegia. Restorative Neurology and Neuroscience, 36(1), 73–82. 29439365 10.3233/RNN-170775 PMC5817907

[ref010] Chen, R. , Cohen, L. G. , Hallett, M. (1997) Role of the ipsilateral motor cortex in voluntary movement. Canadian Journal of Neurological Sciences, 24(4), 284–291. 10.1017/s0317167100032947 9398974

[ref011] Chettouf, S. , Rueda-Delgado, L. M. , de Vries, R. , Ritter, P. , Daffertshofer, A. (2020) Are unimanual movements bilateral? Neuroscience and Biobehavioral Reviews, 113, 39–50. 32142801 10.1016/j.neubiorev.2020.03.002

[ref012] Chiou, S.-Y. , Wang, R.-Y. , Liao, K.-K. , Wu, Y.-T. , Lu, C.-F. , Yang, Y.-R. (2013) Co-activation of primary motor cortex ipsilateral to muscles contracting in a unilateral motor task. Clinical Neurophysiology, 124(7), 1353–1363. 23478202 10.1016/j.clinph.2013.02.001

[ref013] Cramer, S. C. , Finklestein, S. P. , Schaechter, J. D. , Bush, G. , Rosen, B. R. (1999) Activation of distinct motor cortex regions during ipsilateral and contralateral finger movements. Journal of Neurophysiology, 81(1), 383–387. 9914297 10.1152/jn.1999.81.1.383

[ref014] Dassonville, P. , Lewis, S. , Zhu, X.-H. , Uğurbil, K. , Kim, S.-G. , Ashe, J. (1998) Effects of movement predictabilityon cortical motor activation. Neuroscience Research, 32(1), 65–74. 9831253 10.1016/s0168-0102(98)00064-9

[ref015] Dempsey, L. A. , Cooper, R. J. , Roque, T. , Correia, T. , Magee, E. , Powell, S. , Gibson, A. P. , Hebden, J. (2015) Data-driven approach to optimum wavelength selection for diffuse optical imaging. Journal of Biomedical Optics, 20(1), 016003. 25562501 10.1117/1.JBO.20.1.016003

[ref016] Derosiere, G. , Alexandre, F. , Bourdillon, N. , Mandrick, K. , Ward, T. E. , Perrey, S. (2014) Similar scaling of contralateral and ipsilateral cortical responses during graded unimanual force generation. Neuroimage, 85, 471–477. 23416251 10.1016/j.neuroimage.2013.02.006

[ref017] Favre, I. , Zeffiro, T. A. , Detante, O. , Krainik, A. , Hommel, M. , Jaillard, A. (2014) Upper limb recovery after stroke is associated with ipsilesional primary motor cortical activity: a meta-analysis. Stroke, 45(4), 1077–1083. 24525953 10.1161/STROKEAHA.113.003168

[ref018] Heming, E. A. , Cross, K. P. , Takei, T. , Cook, D. J. , Scott, S. H. (2019) Independent representations of ipsilateral and contralaterallimbs in primary motor cortex. Elife, 8, e48190. 31625506 10.7554/eLife.48190 PMC6824843

[ref019] Herold, F. , Wiegel, P. , Scholkmann, F. , Thiers, A. , Hamacher, D. , Schega, L. (2017) Functional near-infrared spectroscopy in movement science: a systematic review on cortical activity in postural and walking tasks. Neurophotonics, 4(4), 041403. 28924563 10.1117/1.NPh.4.4.041403 PMC5538329

[ref020] Horenstein, C. , Lowe, M. J. , Koenig, K. A. , Phillips, M. D. (2009) Comparison of unilateral and bilateral complex finger tapping-related activation in premotor and primary motor cortex. Human Brain Mapping, 30(4), 1397–1412. 18537112 10.1002/hbm.20610 PMC6871138

[ref021] Huang, M. X. , Harrington, D. L. , Paulson, K. M. , Weisend, M. P. , Lee, R. R. (2004) Temporal dynamics of ipsilateral and contralateral motor activity during voluntary finger movement. Human Brain Mapping, 23(1), 26–39. 15281139 10.1002/hbm.20038 PMC6872033

[ref022] Huppert TJ, D. S. , Franceschini MA. , Boas DA. . (2009) HomER: a review of time-series analysis methods for near-infrared spectroscopy of the brain. Applied Optics, 48, D280–298. 19340120 10.1364/ao.48.00d280 PMC2761652

[ref023] Irani, F. , Platek, S. M. , Bunce, S. , Ruocco, A. C. , Chute, D. (2007) Functional near infrared spectroscopy (fNIRS): an emerging neuroimaging technology with important applications for the study of brain disorders. The Clinical Neuropsychologist, 21(1), 9–37. 17366276 10.1080/13854040600910018

[ref024] Johansen-Berg, H. , Rushworth, M. F. , Bogdanovic, M. D. , Kischka, U. , Wimalaratna, S. , Matthews, P. M. (2002) The role of ipsilateral premotor cortex in hand movement after stroke. Proceedings of the National Academy of Sciences, 99(22), 14518–14523. 10.1073/pnas.222536799 PMC137915 12376621

[ref025] Lee, S. H. , Jin, S. H. , An, J. (2019) The difference in cortical activation pattern for complex motor skills: A functional near-infrared spectroscopy study. Scientific Reports, 9(1), 1–9. 31575954 10.1038/s41598-019-50644-9 PMC6773684

[ref026] Leff, D. R. , Orihuela-Espina, F. , Elwell, C. E. , Athanasiou, T. , Delpy, D. T. , Darzi, A. W. , Yang, G.-Z. (2011) Assessment of the cerebral cortex during motor task behaviours in adults: a systematic review of functional near infrared spectroscopy (fNIRS) studies. Neuroimage, 54(4), 2922–2936. 21029781 10.1016/j.neuroimage.2010.10.058

[ref027] Lundell, H. , Christensen, M. S. , Barthélemy, D. , Willerslev-Olsen, M. , Biering-Sørensen, F. , Nielsen, J. B. (2011) Cerebral activation is correlated to regional atrophy of the spinal cord and functional motor disability in spinal cord injured individuals. Neuroimage, 54(2), 1254–1261. 20851198 10.1016/j.neuroimage.2010.09.009

[ref028] Marsman, M. , Wagenmakers, E.-J. (2017) Bayesian benefits with JASP. European Journal of Developmental Psychology, 14(5), 545–555. doi: 10.1080/17405629.2016.1259614

[ref029] MATLAB. (2018) version 9.5.944444 (R2018b). Natick, Massachusetts: The MathWorks Inc.

[ref030] Matsumoto, T. , Watanabe, T. , Kuwabara, T. , Yunoki, K. , Chen, X. , Kubo, N. , Kirimoto, H. (2021) Excitability of the Ipsilateral Primary Motor Cortex During Unilateral Goal-Directed Movement. Frontiers in Human Neuroscience, 15, 77. 10.3389/fnhum.2021.617146 PMC7925409 33679346

[ref031] McDowell, C. L. , Moberg, E. , Smith, A. G. (1979) International conference on surgical rehabilitation of the upper limb in tetraplegia. Journal of Hand Surgery, 4(4), 387–390. 38273 10.1016/s0363-5023(79)80083-0

[ref032] Merzenich, M. M. , Van Vleet, T. M. , Nahum, M. (2014) Brain plasticity-based therapeutics. Frontiers in Human Neuroscience, 8, 385. 25018719 10.3389/fnhum.2014.00385 PMC4072971

[ref033] Nardone, R. , Höller, Y. , Höller, P. , Thon, N. , Thomschewski, A. , Brigo, F. , Trinka, E. (2013) The role of the ipsilateral primary motor cortex in movement control after spinal cord injury: A TMS study. Neuroscience Letters, 552, 21–24. 23880020 10.1016/j.neulet.2013.07.011

[ref034] Perezgonzalez, J. D. (2015) Fisher, Neyman-Pearson or NHST? A tutorial for teaching data testing. Frontiers in Psychology, 6, 223. 25784889 10.3389/fpsyg.2015.00223 PMC4347431

[ref035] Scholkmann, F. , Wolf, M. (2013) General equation for the differential pathlength factor of the frontal human head depending on wavelength and age. Journal of Biomedical Optics, 18(10), 105004. 24121731 10.1117/1.JBO.18.10.105004

[ref036] Schwartz, A. B. (2016) Movement: how the brain communicates with the world. Cell, 164(6), 1122–1135. 26967280 10.1016/j.cell.2016.02.038 PMC4818644

[ref037] Schweiger, M. , Arridge, S. R. (2014) The Toast++software suite for forward and inverse modeling in optical tomography. Journal of Biomedical Optics, 19(4), 040801. 24781586 10.1117/1.JBO.19.4.040801

[ref038] Shibuya, K. , Kuboyama, N. (2007) Human motor cortex oxygenation during exhaustive pinching task. Brain Research, 1156, 120–124. 17543291 10.1016/j.brainres.2007.05.009

[ref039] Shibuya, K. , Sadamoto, T. , Sato, K. , Moriyama, M. , Iwadate, M. (2008) Quantification of delayed oxygenation in ipsilateral primary motor cortex compared with contralateral side during a unimanual dominant-hand motor task using near-infrared spectroscopy. Brain Research, 1210, 142–147. 18423579 10.1016/j.brainres.2008.03.009

[ref040] Steinbrink, J. , Villringer, A. , Kempf, F. , Haux, D. , Boden, S. , Obrig, H. (2006) Illuminating the BOLD signal: combined fMRI–fNIRS studies. Magnetic Resonance Imaging, 24(4), 495–505. 16677956 10.1016/j.mri.2005.12.034

[ref041] Van Dokkum, L. E. , Le Bars, E. , Mottet, D. , Bonafé, A. , Menjot de Champfleur, N. , Laffont, I. (2018) Modified Brain Activations of the Nondamaged Hemisphere During Ipsilesional Upper-Limb Movement in Persons With Initial Severe Motor Deficits Poststroke. Neurorehabilitation and Neural Repair, 32(1), 34–45. 29276841 10.1177/1545968317746783

[ref042] Verstynen, T. , Diedrichsen, J. , Albert, N. , Aparicio, P. , Ivry, R. B. (2005) Ipsilateral motor cortex activity during unimanual hand movements relates to task complexity. Journal of Neurophysiology, 93(3), 1209–1222. 15525809 10.1152/jn.00720.2004

[ref043] Wangdell, J. , Bunketorp-Käll, L. , Koch-Borner, S. , Fridén, J. (2016) Early active rehabilitation after grip reconstructive surgery in tetraplegia. Archives of Physical Medicine and Rehabilitation, 97(6), S117–S125. 27233586 10.1016/j.apmr.2015.09.025

[ref044] Werhahn, K. J. , Conforto, A. B. , Kadom, N. , Hallett, M. , Cohen, L. G. (2003) Contribution of the ipsilateral motor cortex to recovery after chronic stroke. Annals of Neurology: Official Journal of the American Neurological Association and the Child Neurology Society, 54(4), 464–472. 10.1002/ana.10686 14520658

[ref045] Wilkins, K. B. , Yao, J. (2020) Coordination of multiple joints increases bilateral connectivity with ipsilateral sensorimotor cortices. Neuroimage, 207, 116344. 31730924 10.1016/j.neuroimage.2019.116344 PMC7192312

[ref046] Wisneski, K. J. , Anderson, N. , Schalk, G. , Smyth, M. , Moran, D. , Leuthardt, E. C. (2008) Unique cortical physiology associated with ipsilateral hand movements and neuroprosthetic implications. Stroke, 39(12), 3351–3359. 18927456 10.1161/STROKEAHA.108.518175

[ref047] Yang, C.-L. , Lim, S. B. , Peters, S. , Eng, J. J. (2020) Cortical Activation During Shoulder and Finger Movements in Healthy Adults: A Functional Near-Infrared Spectroscopy (fNIRS) Study. Frontiers in Human Neuroscience, 14, 260. 32733221 10.3389/fnhum.2020.00260 PMC7362764

[ref048] Ye, J. C. , Tak, S. , Jang, K. E. , Jung, J. , Jang, J. (2009) NIRS-SPM: statistical parametric mapping for near-infrared spectroscopy. Neuroimage, 44(2), 428–447. 18848897 10.1016/j.neuroimage.2008.08.036

[ref049] Yokoyama, N. , Ohtaka, C. , Kato, K. , Kubo, H. , Nakata, H. (2019) The difference in hemodynamic responses between dominant and non-dominant hands during muscle contraction and relaxation: An fNIRS study. PloS One, 14(7), e0220100. 31323051 10.1371/journal.pone.0220100 PMC6641204

